# Association of Mineralocorticoid Receptor Antagonist Use With All-Cause Mortality and Hospital Readmission in Older Adults With Acute Decompensated Heart Failure

**DOI:** 10.1001/jamanetworkopen.2019.5892

**Published:** 2019-06-21

**Authors:** Hidenori Yaku, Takao Kato, Takeshi Morimoto, Yasutaka Inuzuka, Yodo Tamaki, Neiko Ozasa, Erika Yamamoto, Yusuke Yoshikawa, Takeshi Kitai, Ryoji Taniguchi, Moritake Iguchi, Masashi Kato, Mamoru Takahashi, Toshikazu Jinnai, Tomoyuki Ikeda, Kazuya Nagao, Takafumi Kawai, Akihiro Komasa, Ryusuke Nishikawa, Yuichi Kawase, Takashi Morinaga, Mamoru Toyofuku, Yuta Seko, Yutaka Furukawa, Yoshihisa Nakagawa, Kenji Ando, Kazushige Kadota, Satoshi Shizuta, Koh Ono, Yukihito Sato, Koichiro Kuwahara, Takeshi Kimura

**Affiliations:** 1 Department of Cardiovascular Medicine, Kyoto University Graduate School of Medicine, Kyoto, Japan; 2 Department of Clinical Epidemiology, Hyogo College of Medicine, Hyogo, Japan; 3 Department of Cardiovascular Medicine, Shiga General Hospital, Shiga, Japan; 4 Division of Cardiology, Tenri Hospital, Nara, Japan; 5 Department of Cardiovascular Medicine, Kobe City Medical Center General Hospital, Hyogo, Japan; 6 Department of Cardiology, Hyogo Prefectural Amagasaki General Medical Center, Hyogo, Japan; 7 Department of Cardiology, National Hospital Organization Kyoto Medical Center, Kyoto, Japan; 8 Department of Cardiology, Mitsubishi Kyoto Hospital, Kyoto, Japan; 9 Department of Cardiology, Shimabara Hospital, Kyoto, Japan; 10 Department of Cardiology, Japanese Red Cross Otsu Hospital, Shiga, Japan; 11 Department of Cardiology, Hikone Municipal Hospital, Shiga, Japan; 12 Department of Cardiology, Osaka Red Cross Hospital, Osaka, Japan; 13 Department of Cardiology, Shizuoka General Hospital, Shizuoka, Japan; 14 Department of Cardiology, Kurashiki Central Hospital, Okayama, Japan; 15 Department of Cardiology, Kokura Memorial Hospital, Fukuoka, Japan; 16 Department of Cardiology, Japanese Red Cross Wakayama Medical Center, Wakayama, Japan; 17 Department of Cardiovascular Medicine, Shiga University Graduate School of Medicine, Shiga, Japan; 18 Department of Cardiovascular Medicine, Shinshu University Graduate School of Medicine, Nagano, Japan

## Abstract

**Importance:**

Scarce data are available on the association of mineralocorticoid receptor antagonist (MRA) use with outcomes in acute decompensated heart failure (ADHF).

**Objective:**

To investigate the association of MRA use with all-cause mortality and hospital readmission in patients with ADHF.

**Design, Setting, and Participants:**

This cohort study examines participants enrolled in the Kyoto Congestive Heart Failure (KCHF) registry, a physician-initiated, prospective, multicenter cohort study of consecutive patients admitted for ADHF, between October 1, 2014, and March 31, 2016, into 1 of 19 secondary and tertiary hospitals throughout Japan. To balance the baseline characteristics associated with the selection of MRA use, a propensity score–matched cohort design was used, yielding 2068 patients. Data analysis was conducted from April to August 2018.

**Exposures:**

Prescription of MRA at discharge from the index hospitalization.

**Main Outcomes and Measures:**

Composite of all-cause death or heart failure hospitalization after discharge.

**Results:**

Among 3717 patients hospitalized for ADHF, 1678 patients (45.1%) had received MRA at discharge and 2039 (54.9%) did not. After propensity score matching, 2068 patients (with a median [interquartile range] age of 80 [72-86] years, and of whom 937 [45.3%] were women) were included. In the matched cohort (n = 1034 in each group), the cumulative 1-year incidence of the primary outcome was statistically significantly lower in the MRA use group than in the no MRA use group (28.4% vs 33.9%; hazard ratio [HR], 0.81; 95% CI, 0.70-0.93; *P* = .003). Of the components of the primary outcome, the cumulative 1-year incidence of heart failure hospitalization was significantly lower in the MRA use group than in the no MRA use group (18.7% vs 24.8%; HR, 0.70; 95% CI, 0.60-0.86; *P* < .001), whereas no difference in mortality was found between the 2 groups (15.6% vs 15.8%; HR, 0.98; 95% CI, 0.82-1.18; *P* = .85). No difference in all-cause hospitalization was observed between the 2 groups (35.3% vs 38.2%; HR, 0.88; 95% CI, 0.77-1.01; *P* = .07). In additional analyses that stratified by left ventricular ejection fraction, the association of MRA use with the primary outcome was statistically significant in patients with left ventricular ejection fraction of 40% or greater.

**Conclusions and Relevance:**

Use of MRA at discharge from ADHF hospitalization did not appear to be associated with lower mortality but was associated with a lower risk of heart failure readmission. This finding suggests that MRA treatment at discharge may have minimal, if any, clinical advantages.

## Key Points

**Question:**

Is use of mineralocorticoid receptor antagonist at discharge associated with better outcomes in patients hospitalized for acute decompensated heart failure?

**Findings:**

In this cohort study of 2068 propensity score–matched Japanese patients hospitalized for acute decompensated heart failure, mineralocorticoid receptor antagonist administered at discharge was statistically significantly associated with a lower risk for the primary composite outcome of mortality or heart failure readmission, although no difference in all-cause death was observed.

**Meaning:**

Use of mineralocorticoid receptor antagonist at discharge from acute decompensated heart failure hospitalization may be associated with heart failure hospitalization but not with lower mortality.

## Teaser

This cohort study of Japanese participants with acute decompensated heart failure in the Kyoto Congestive Heart Failure study evaluates the association of mineralocorticoid receptor antagonist use with all-cause mortality and hospital readmission.

## Introduction

Mineralocorticoid receptor antagonists (MRAs), such as spironolactone and eplerenone, have been associated with reductions in mortality in patients with stable chronic heart failure with reduced ejection fraction (HFrEF).^1,2^ In patients with stable heart failure with preserved ejection fraction (HFpEF), a randomized clinical trial (RCT) has suggested that MRA is associated with reductions in heart failure hospitalization, although the study did not meet the primary composite end point of death from cardiovascular causes, aborted cardiac arrest, or heart failure hospitalization.^3,4^

In contrast, scarce data are available on the long-term outcomes of MRA use after discharge of patients hospitalized for acute decompensated heart failure (ADHF).^5^ Because these patients had experienced the acute exaggeration of heart failure and hospital admission, they had a high risk for cardiac mortality and rehospitalization owing to worsening of heart failure.^6^ In addition, the contemporary patient population hospitalized for ADHF might be substantially different from stable patients with heart failure who are enrolled in RCTs. The role of MRA in postdischarge management needs to be investigated in patients with ADHF and multiple comorbidities. Therefore, we sought to explore the association between MRA administered at discharge from ADHF hospitalization and clinical outcomes using the registry of a large contemporary all-comer study (Kyoto Congestive Heart Failure [KCHF]) in Japan of patients with ADHF hospitalization.

## Methods

### Study Design, Setting, and Population

The KCHF is a physician-initiated, prospective, multicenter cohort study that enrolled consecutive patients who were hospitalized for ADHF for the first time between October 1, 2014, and March 31, 2016. These patients were admitted into 19 secondary and tertiary hospitals, including rural and urban as well as large and small institutions, throughout Japan. The study protocol was approved by the institutional review board of each participating hospital. A waiver of written informed consent from each patient was granted by the institutional review boards of Kyoto University and each participating center because the study met the conditions of the Japanese ethical guidelines for epidemiological study and the US policy for protecting human research participants.^7,8^ This study followed the Strengthening the Reporting of Observational Studies in Epidemiology (STROBE) reporting guideline.

The details of the KCHF study design and patient enrollment are described elsewhere.^9,10^ Briefly, we enrolled all patients with ADHF, as defined by the modified Framingham criteria, who were admitted to the participating hospitals and patients who underwent heart failure–specific treatment involving intravenous drugs within 24 hours after hospital presentation. Patient records were anonymized before analysis. Data analysis was conducted from April 2018 to August 2018.

Among the 4056 enrolled patients in the KCHF registry, 3785 patients (93.3%) were discharged alive after hospitalization for ADHF. Clinical follow-up data were collected in October 2017. The attending physicians or research assistants at each participating facility collected clinical events data after the index hospitalization from hospital medical records or from patients, their relatives, or their referring physicians (with patient consent).

**Figure 1.**
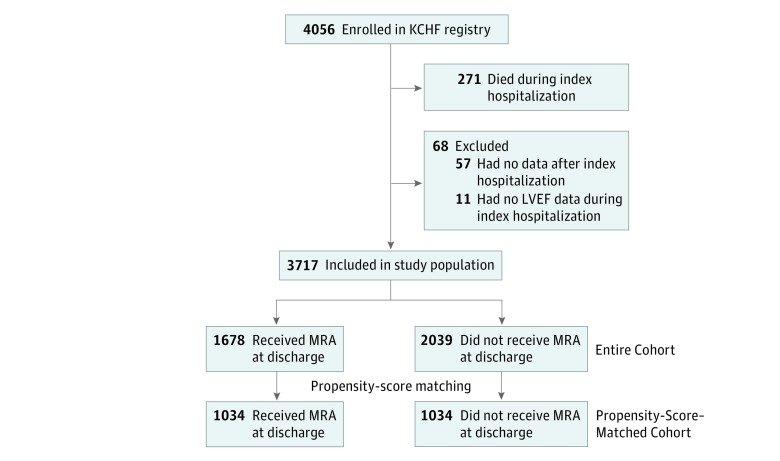
Study Flowchart KCHF indicates Kyoto Congestive Heart Failure; LVEF, left ventricular ejection fraction; and MRA, mineralocorticoid receptor antagonist.

In the present study, we compared the clinical outcomes between patients who received MRA at discharge and those who did not receive it. We excluded 11 patients for missing data on left ventricular ejection fraction (LVEF) during the index hospitalization to stratify the analysis according to LVEF. After further exclusions of 57 patients without any follow-up data after discharge, the study population consisted of 3717 patients with known LVEF and postdischarge clinical follow-up data (Figure 1).

### Definitions

We defined the use of MRA (MRA use group) as any new or continued prescription of spironolactone or eplerenone at discharge from the index hospitalization. The detailed definitions of baseline clinical characteristics have been described previously.^10^

The primary outcome measure was a composite of all-cause death or heart failure hospitalization after discharge from the index hospitalization. Other outcome measures included heart failure hospitalization, all-cause death, cardiovascular death, sudden death, and any-cause hospitalization. Death was regarded as cardiovascular in origin unless obvious noncardiovascular causes could be identified. Cardiovascular death included death related to heart failure, sudden death, death related to stroke, and death from other cardiovascular causes. Sudden death was the unexplained death in a previously stable patient. Stroke was either ischemic or hemorrhagic that required either acute or prolonged hospitalization and had symptoms that lasted more than 24 hours. Heart failure hospitalization was due to worsening of heart failure, requiring intravenous drug therapy.^9^ Heart failure was classified according to baseline LVEF as with reduced LVEF (<40%) or with preserved LVEF (≥40%).

Laboratory tests were performed on patient admission. Missing laboratory values are presented in eTable 1 in the Supplement.

### Statistical Analysis

Categorical variables were presented as numbers with percentages and were compared with the χ^2^ test. Continuous variables were expressed as means with SDs or medians with interquartile range and were compared with an unpaired, 2-tailed *t* test when normally distributed or with Wilcoxon rank sum test when not normally distributed. Cumulative incidences were estimated by the Kaplan-Meier method and compared using the log-rank test. To account for the competing risk of all-cause death, we also calculated cumulative incidence functions of heart failure hospitalization and compared the differences between the 2 groups by Gray test in the matched cohort.^11,12^

We regarded the date of discharge as time 0 for clinical follow-up. We compared baseline characteristics with clinical outcomes on the basis of the presence or absence of the use of MRA at discharge from the index hospitalization. To balance the baseline characteristics associated with the selection of MRA use, we used a propensity score–matched cohort design as the main analysis. We also performed analysis in the entire cohort as the sensitivity analysis to explore the robustness of the findings. We compared groups by intention-to-treat analysis, regardless of the discontinuation of MRA during follow-up.

A logistic regression model was developed to make the propensity score for the choice of MRA with 16 baseline variables that were clinically relevant to the choice of MRA treatment (Table 1). Based on the estimated propensity score, patients in the group who did not receive MRA treatment (no MRA use) were matched with those in the group who received MRA treatment (MRA use) by using a 1:1 greedy matching technique.^13^ We compared the baseline characteristics and evaluated the cumulative incidences using the propensity score–matched cohort. We estimated the hazard ratios (HRs) and 95% CIs with Cox proportional hazards regression model. We conducted subgroup analyses stratified by LVEF (<40% or ≥40%) and other clinically relevant factors in the Cox models. We assessed the interactions between the subgroup factors and the associations of MRA use in the Cox models. For the sensitivity analysis using the entire cohort, MRA and the 25 clinically relevant risk-adjusting variables were simultaneously included in the Cox models (Table 1). The continuous variables were dichotomized by clinically meaningful reference values or median values. We expressed the association of the MRA use group with the no MRA use group with all of the outcome measures as HRs with 95% CIs.

**Table 1.** Patient Characteristics of the Study Population in the Matched and Entire Cohort

Variable	No. (%)					
	Propensity Score-Matched Cohort			Entire Cohort		
	MRA Use (n = 1034)	No MRA Use (n = 1034)	*P* Value	MRA Use (n = 1678)	No MRA Use (n = 2039)	*P* Value
**Clinical Characteristic**						
Age, median (IQR), y	80 (72-86)	80 (73-87)	.37	79 (70-85)	81 (73-87)	<.001
Age ≥80 y^a^^,^^b^	536 (52)	534 (52)	.93	793 (47)	1135 (56)	<.001
Female sex^a^^,^^b^	469 (45)	468 (45)	.96	763 (45)	905 (44)	.51
BMI, mean (SD)	23.0 (4.7)	22.8 (4.3)	.26	23.0 (4.8)	22.8 (4.2)	.20
BMI ≤22^b^	449 (46)	458 (46)	.92	744 (47)	893 (46)	.57
Origin						
Ischemic heart disease	335 (32)	323 (31)	.57	530 (32)	675 (33)	.31
ACS^b^	61 (5.9)	58 (5.6)	.78	86 (5.1)	119 (5.8)	.34
Hypertensive heart disease	260 (25)	269 (26)	.65	368 (22)	559 (27)	<.001
Cardiomyopathy	159 (15)	144 (14)	.35	318 (19)	238 (12)	<.001
Valvular heart disease	200 (19)	210 (20)	.58	338 (20)	397 (19)	.61
Other heart disease	80 (7.7)	88 (8.5)	.52	124 (7.4)	170 (8.3)	.29
Medical history						
Previous HF hospitalization^a^^,^^b^	314 (30)	299 (29)	.47	549 (33)	768 (39)	<.001
Atrial fibrillation or flutter^b^	418 (40)	447 (43)	.20	714 (43)	836 (41)	.34
Hypertension^a^^,^^b^	758 (73)	761 (74)	.88	1135 (68)	1555 (76)	<.001
Diabetes^a^^,^^b^	378 (37)	348 (34)	.17	595 (35)	797 (39)	.02
Dyslipidemia	380 (37)	413 (40)	.14	612 (36)	840 (41)	.003
Previous myocardial infarction^a^^,^^b^	225 (22)	223 (22)	.92	366 (22)	470 (23)	.37
Previous stroke^b^	161 (16)	139 (13)	.17	248 (15)	342 (17)	.10
Previous PCI or CABG	248 (24)	250 (24)	.92	392 (23)	561 (28)	.004
Current smoking^b^	131 (13)	129 (13)	.89	222 (13)	230 (12)	.08
VT or VF	44 (4.3)	41 (4.0)	.74	86 (5.1)	68 (3.3)	.007
Chronic kidney disease	397 (38)	418 (40)	.35	604 (36)	1033 (51)	<.001
Chronic lung disease^b^	122 (12)	138 (13)	.29	203 (12)	285 (14)	.09
Malignant neoplasm	153 (15)	143 (14)	.56	234 (14)	301 (15)	.47
Dementia	187 (18)	177 (17)	.56	281 (17)	373 (18)	.21
Social background						
Poor medical adherence	183 (18)	162 (16)	.22	293 (17)	335 (16)	.40
With occupation	146 (14)	128 (12)	.24	261 (16)	233 (11)	<.001
Daily life activities						
Ambulatory^b^	833 (81)	818 (80)	.57	1352 (81)	1589 (79)	.04
Use of wheelchair, outdoor only	66 (6.4)	82 (8.0)	.16	109 (6.6)	165 (8.2)	.06
Use of wheelchair, outdoor and indoor	92 (8.9)	91 (8.9)	.97	141 (8.5)	195 (9.7)	.22
Bedridden	39 (3.8)	33 (3.2)	.49	59 (3.6)	70 (3.4)	.89
**Vital Signs at Presentation**						
BP, mm Hg						
Systolic, mean (SD)	149 (34)	149 (34)	.91	145 (34)	151 (36)	<.001
<90^a^^,^^b^	19 (1.8)	25 (2.4)	.36	48 (2.9)	47 (2.3)	.29
Diastolic, mean (SD)	87 (24)	85 (24)	.25	85 (24)	85 (24)	.53
Heart rate, mean (SD), bpm	98 (27)	97 (29)	.93	97 (27)	95 (28)	.005
<60 bpm^b^	51 (4.9)	72 (6.9)	.051	87 (5.2)	163 (8.1)	<.001
Rhythms at presentation						
Sinus rhythm	582 (56)	583 (56)	.96	897 (53)	1173 (58)	.01
Atrial fibrillation or flutter	376 (36)	386 (37)	.65	652 (39)	703 (34)	.006
NYHA class III or IV^a^^,^^b^	904 (87)	913 (88)	.54	1456 (87)	1766 (87)	.64
Tests at admission						
LVEF, mean (SD), %	46 (16)	47 (16)	.11	44 (16)	48 (16)	<.001
HFrEF (EF <40%)^a^^,^^b^	368 (36)	370 (36)	.93	722 (43)	661 (32)	<.001
BNP, median (IQR), pg/mL	699 (381-1228)	699 (402-1218)	.71	700 (381-1216)	721 (403-1287)	.20
NT-proBNP, median (IQR), pg/mL	4640 (2189-9690)	5530 (2947-9692)	.14	4810 (2427-10 773)	6405 (3008-14 109)	.003
Serum creatinine, median (IQR), mg/dL	1.0 (0.7-1.3)	1.1 (0.8-1.4)	<.001	1.0 (0.8-1.3)	1.3 (0.9-1.9)	<.001
eGFR, mean (SD), mL/min/1.73m^2^	50 (35-67)	45 (33-59)	<.001	51 (37-67)	38 (24-55)	<.001
<30 mL/min/1.73m^2^^a^^,^^b^	189 (18)	186 (18)	.86	253 (15)	725 (36)	<.001
Blood urea nitrogen, median (IQR), mg/dL	22 (16-30)	23 (18-32)	.001	21 (16-29)	26 (19-39)	<.001
Albumin, mean (SD), g/dL	3.5 (0.5)	3.5 (0.5)	.74	3.5 (0.5)	3.5 (0.5)	.04
<3.0 g/dL^b^	130 (13)	122 (12)	.54	210 (13)	270 (14)	.52
Sodium, mean (SD), mEq/L	139 (4.3)	139 (4.1)	.34	139 (4.3)	139 (4.1)	.16
<135 mEq/L^b^	131 (13)	101 (9.8)	.04	222 (13)	211 (10)	.007
Potassium, mean (SD), mEq/L	4.1 (0.6)	4.2 (0.6)	.003	4.1 (0.6)	4.3 (0.7)	<.001
≥5.0 mEq/L^a^	96 (9.3)	108 (10)	.38	135 (8.1)	297 (15)	<.001
Hemoglobin, mean (SD), g/dL	11.8 (2.3)	11.6 (2.3)	.18	11.9 (2.4)	11.2 (2.3)	<.001
Anemia, No. (%)^a^^,^^b^	665 (64)	670 (65)	.82	995 (59)	1462 (72)	<.001
MRA before the index admission^a^^,^^b^	117 (11)	112 (11)	.73	522 (31)	129 (6.3)	<.001
Medications at discharge						
ACEI/ARB and β-blocker	464 (45)	463 (45)	.96	819 (49)	742 (36)	<.001
ACEI or ARB^a^^,^^b^	625 (60)	627 (61)	.93	1051 (63)	1086 (53)	<.001
β-Blocker^a^^,^^b^	702 (68)	703 (68)	.96	1203 (72)	1266 (62)	<.001
Loop diuretics^a^^,^^b^	911 (88)	908 (88)	.84	1541 (92)	1474 (72)	<.001
Thiazide	40 (3.9)	63 (6.1)	.02	73 (4.4)	145 (7.1)	<.001
Tolvaptan	97 (9.4)	92 (8.9)	.70	176 (10)	214 (11)	.99
Digoxin	59 (5.7)	58 (5.6)	.92	127 (7.6)	84 (4.1)	<.001
Warfarin sodium	236 (23)	242 (23)	.75	420 (25)	504 (25)	.83
DOAC	230 (22)	244 (24)	.46	379 (23)	381 (19)	.003

Abbreviations: ACEI, angiotensin-converting enzyme inhibitor; ACS, acute coronary syndrome; ARB, angiotensin-receptor blocker; BMI, body mass index (calculated as weight in kilograms divided by height in meters squared); BNP, brain-type natriuretic peptide; BP, blood pressure; CABG, coronary artery bypass graft; DOAC, direct oral anticoagulant; EF, ejection fraction; eGFR, estimated glomerular filtration rate; HF, heart failure; HFrEF, heart failure with reduced ejection fraction; IQR, interquartile range; LVEF, left ventricular ejection fraction; MRA, mineralocorticoid receptor antagonist; NT-proBNP, N-terminal-proBNP; NYHA, New York Heart Association; PCI, percutaneous coronary intervention; VF, ventricular fibrillation; VT, ventricular tachycardia.

SI conversion factors: To convert albumin to grams per liter, multiply by 10; blood urea nitrogen level to millimoles per liter, multiply by 0.357; BNP to nanograms per liter, multiply by 1.0; eGFR to milliliters per second per meters squared, multiply by 0.0167; hemoglobin level to grams per liter, multiply by 10.0; potassium level to millimoles per liter, multiply by 1.0; serum creatinine to micromoles per liter, multiply by 88.4; and sodium to millimoles per liter, multiply by 1.0.

^a^ Variables relevant to the choice of MRA were selected for logistic regression model for developing a propensity score for the choice of MRA.

^b^ Risk-adjusting variables were selected for Cox proportional hazard models in the unmatched cohort.

To focus the association of LVEF, we performed post hoc analyses. First, we stratified the entire cohort into the 2 strata by LVEF (<40% or ≥40%) and calculated the propensity score for the choice of MRA use in each LVEF stratum. Then, we generated the propensity score–matched cohort in the same fashion as the main analysis and compared the differences between the 2 groups in each LVEF stratum.

All statistical analyses were conducted by 2 of our physicians (H.Y. and Y.Y.) and our statistician (T. Morimoto) using JMP, version 13.0, and SAS, version 9.4 (SAS Institute Inc) or R (R Project for Statistical Computing). Two-tailed *P* < .05 was considered statistically significant.

## Results

### Patient Characteristics

In the study population of 3717 patients, 1678 (45.1%) patients had received MRA treatment at discharge and 2039 (54.9%) did not. The MRA treatment included spironolactone (median dose, 25 mg) in 1570 patients (93.6%) and eplerenone (median dose, 50 mg) in 108 patients (6.4%). Regarding the baseline clinical characteristics before matching, the patients in the MRA use group were younger and had a lower prevalence of previous heart failure hospitalization, hypertension, diabetes, dyslipidemia, previous percutaneous coronary intervention or coronary artery bypass graft, renal dysfunction, and anemia (Table 1). No significant differences in body mass index, atrial fibrillation (AF) or atrial flutter (AFL), previous myocardial infarction, previous stroke, chronic lung disease, malignant neoplasm, and dementia were observed between the 2 groups (Table 1). The MRA use group was more likely to have a hypertensive origin and AF or AFL at presentation, higher heart rate, lower blood pressure, lower levels of blood urea nitrogen and potassium, and a reduced LVEF (Table 1). Regarding medical treatment at discharge, angiotensin-converting enzyme inhibitor or angiotensin receptor blocker, β-blocker, and loop diuretics were more often prescribed in the MRA use group (Table 1).

The propensity score matching yielded a total of 2068 patients: 1034 patients in the MRA use group were matched to 1034 reference patients in the no MRA use group (main cohort: median [IQR] age, 80 [72-86] years; 937 [45.3%] women; mean [SD] LVEF, 46.7% [16.0%]). In the matched cohort, baseline characteristics were well balanced between the 2 groups, except for the slightly but statistically significantly better renal function in the MRA use group than in the no MRA use group (Table 1).

### Clinical Outcomes in the Matched and Entire Cohort

**Figure 2.**
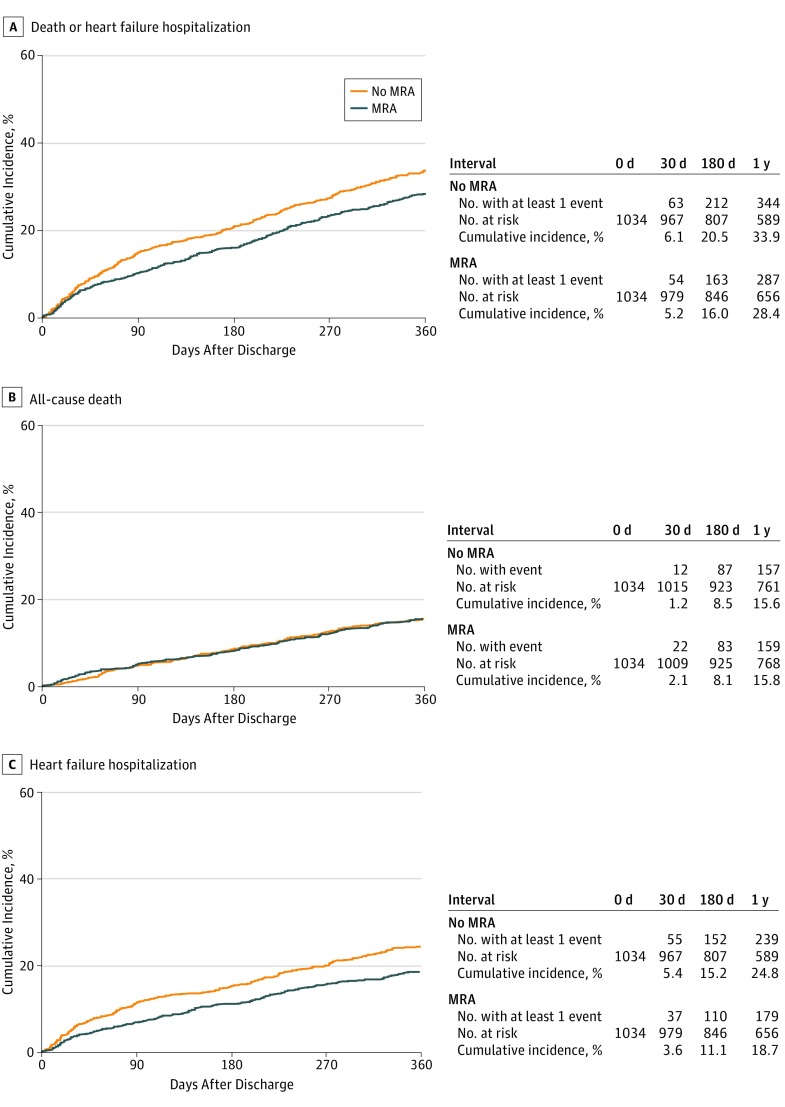
Cumulative Incidence Rates of the Primary Outcome Measure in the Propensity Score–Matched Cohort Log-rank *P* = .003 (A), *P* = .85 (B), and *P* < .001 (C). MRA indicates mineralocorticoid receptor antagonist.

The median (interquartile range [IQR]) length of follow-up was 470 days (357-649 days), with a 96% follow-up rate at 1 year. In the propensity score–matched cohort, the cumulative 1-year incidence of the primary outcome measure (a composite of all-cause death or heart failure hospitalization) in the MRA use group was statistically significantly lower compared with the no MRA use group (28.4% vs 33.9%; HR, 0.81; 95% CI, 0.70-0.93; *P* = .003) (Figure 2A). The cumulative 1-year incidence of all-cause death was not statistically significantly different between the 2 groups (15.6% vs 15.8%; HR, 0.98; 95% CI, 0.82-1.18; *P* = .85) (Figure 2B and Table 2), whereas the cumulative 1-year incidence of heart failure hospitalization in the MRA use group was statistically significantly lower than that in the no MRA use group (18.7% vs 24.8%; HR, 0.70; 95% CI, 0.60-0.86; *P* < .001) (Figure 2C and Table 2). The result was consistent with the result derived from the cumulative incidence function curves accounting for the competing risk of death by Gray test (eFigure 1 in the Supplement).

**Table 2.** Clinical Outcomes in the Matched and Entire Cohort

Outcome	Propensity Score–Matched Cohort				Entire Cohort					
	No. (%)^a^		HR (95% CI)	*P* Value	No. (%)^a^		HR (95% CI)	*P* Value	Adjusted HR (95% CI)^b^	*P* Value
	MRA Use (n = 1034)	No MRA Use (n = 1034)			MRA Use (n = 1678)	No MRA Use (n = 2039)				
Composite of all-cause death or HF hospitalization	287 (28.4)	344 (33.9)	0.81 (0.70-0.93)	.003	503 (30.6)	739 (36.8)	0.79 (0.71-0.88)	<.001	0.81 (0.71-0.92)	.001
HF hospitalization	179 (18.7)	239 (24.8)	0.70 (0.60-0.86)	<.001	335 (21.5)	494 (26.2)	0.77 (0.68-0.88)	<.001	0.74 (0.63-0.87)	<.001
All-cause death	157 (15.6)	159 (15.8)	0.98 (0.82-1.18)	.85	257 (15.7)	367 (18.4)	0.83 (0.72-0.95)	.007	0.93 (0.78-1.11)	.42
Cardiovascular death	96 (9.8)	93 (9.4)	1.07 (0.84-1.36)	.59	155 (9.8)	217 (11.3)	0.88 (0.74-1.05)	.16	1.03 (0.82-1.29)	.80
Sudden death	23 (2.5)	18 (1.9)	1.59 (0.93-2.78)	.09	33 (2.2)	43 (2.3)	1.09 (0.73-1.61)	.68	1.51 (0.92-2.45)	.10
Any hospitalization	344 (35.3)	375 (38.2)	0.88 (0.77-1.01)	.07	580 (36.6)	779 (40.8)	0.85 (0.77-0.94)	.002	0.84 (0.74-0.96)	.007

Abbreviations: HF, heart failure; HR, hazard ratio; MRA, mineralocorticoid receptor antagonist.

^a^ Number of patients with at least 1 event reported as cumulative 1-year incidence, counted through the entire follow-up period.

^b^ Adjusted for the clinically relevant variables described in Table 1.

**Figure 3.**
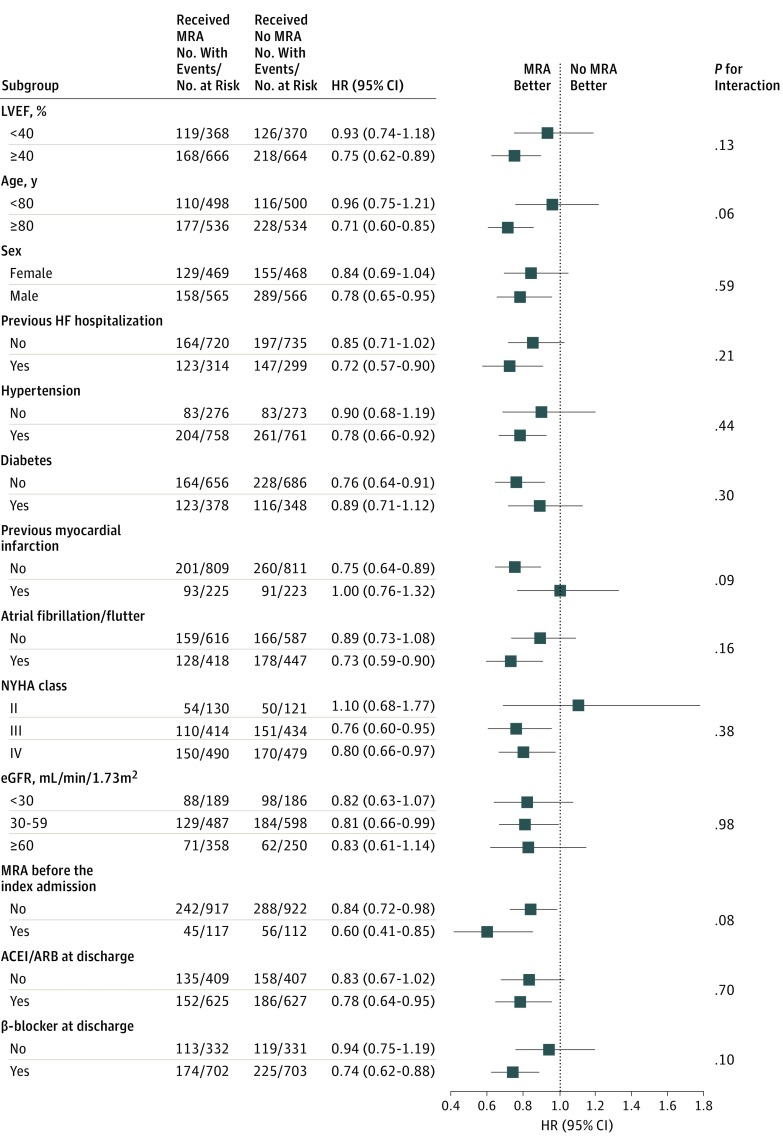
Subgroup Analysis for the Primary Outcome Measure in the Propensity Score–Matched Cohort ACEI indicates angiotensin-converting enzyme inhibitor; ARB, angiotensin-receptor blocker; eGFR, estimated glomerular filtration rate; HF, heart failure; HR, hazard ratio; LVEF, left ventricular ejection fraction; MRA, mineralocorticoid receptor antagonist; and NYHA, New York Heart Association.

In the subgroup analysis (Figure 3), no statistically significant interaction was observed between the HRs for the primary outcome measure associated with the use of MRA and the clinically relevant subgroup factors such as LVEF, age, sex, previous heart failure hospitalization, diabetes, myocardial infarction, AF or AFL, New York Heart Association class, estimated glomerular filtration rate, and use of the antagonists of the renin-angiotensin system and β-blocker (eTable 2 and eTable 3 in the Supplement). Nevertheless, the association of MRA use with the primary outcome measure was greater in patients with HFpEF (25.9% vs 33.4%; HR, 0.75; 95% CI, 0.62-0.89; *P* = .001) than in those with HFrEF (33.1% vs 34.7%; HR, 0.93; 95% CI, 0.74-1.18; *P* = .56) (Figure 3; eFigure 2 in the Supplement).

In the entire cohort, the cumulative 1-year incidence of the primary outcome measure in the MRA use group was statistically significantly lower than that in the no MRA use group (30.6% vs 36.8%; *P* < .001) (eFigure 3 in the Supplement). After adjusting for baseline characteristics, the favorable association of the MRA use group compared with the no MRA use group remained statistically significant (adjusted HR, 0.81; 95% CI, 0.71-0.92; *P* = .001) (Table 2). The results in the entire cohort for the secondary outcome measures, including all-cause death and heart failure hospitalization, were consistent with those in the propensity score–matched cohort (Table 2; eFigure 3 in the Supplement). No difference in any unexpected hospitalization was observed between the 2 groups in the propensity score–matched cohort (35.3% vs 38.2%; HR, 0.88; 95% CI, 0.77-1.01; *P* = .07), although the cumulative 1-year incidence of any unexpected hospitalization in the MRA use group was statistically significantly lower than that in the no MRA use group in the entire cohort (36.6% vs 40.8%; adjusted HR, 0.84; 95% CI, 0.74-0.96; *P* = .007) (Table 2). The results in the entire cohort for the subgroup analysis stratified by LVEF (eFigure 4 and eTable 4 in the Supplement) were consistent with those in the propensity score–matched cohort.

### Post Hoc Analyses

Of the population of 3717 patients, 1383 (37.2%) showed reduced LVEF, and 2334 (62.8%) showed preserved LVEF (eFigure 5 in the Supplement). Propensity score matching resulted in a total of 385 patients with HFrEF and 690 patients with HFpEF for the MRA use group or no MRA use group (eFigure 5, eTable 5, and eTable 6 in the Supplement). In the matched cohort of patients with HFrEF, the cumulative 1-year incidence of the primary outcome measure was not statistically significantly different between the 2 groups (34.7% vs 33.7%; HR, 0.87; 95% CI, 0.78-1.21; *P* = .78) (eFigure 6 in the Supplement). In the matched cohort of patients with HFpEF, the cumulative 1-year incidence of the primary outcome measure in the MRA use group was statistically significantly lower than that in the no MRA use group (26.8% vs 33.7%; HR, 0.78; 95% CI, 0.66-0.93; *P* = .005) (eFigure 6 in the Supplement). The results were consistent with the main results (eFigure 2 in the Supplement) in the propensity score–matched cohort, including patients with HFrEF or HFpEF.

## Discussion

The main findings of the present study were as follows. First, the use of MRA at hospital discharge was associated with a lower risk for the primary outcome measure (a composite of all-cause death or heart failure hospitalization) in patients hospitalized for ADHF; however, MRA use did not seem to be associated with lower mortality but was associated with heart failure hospitalization. Second, when patients were stratified by LVEF at first, some advantages appeared to have potentially accrued to patients with HFpEF.

The clinical utility of MRA in stable patients with heart failure and reduced LVEF was first demonstrated in the Randomized Aldactone Evaluation Study (RALES) in 1991.^1^ Subsequently, a line of evidence emerged that MRA treatment (spironolactone or eplerenone) was associated with reduced morbidity and mortality in patients with HFrEF.^2,14^ The Treatment of Preserved Cardiac Function Heart Failure With an Aldosterone Antagonist (TOPCAT) trial^15^ in patients with stable HFpEF suggested the reduction of heart failure hospitalization with MRA treatment, although the study failed to show the evidence for the mortality advantage of MRA treatment during the median follow-up of 3.3 years. However, to our knowledge, no previous RCT investigated the association of MRA use in patients hospitalized for ADHF with the postdischarge outcomes. Acute decompensated heart failure has the potential risk for decline and eventually in-hospital death. Even during the recovery phase, a certain proportion of patients hospitalized for ADHF are hemodynamically unstable, often with worsening renal function. Therefore, the optimal management of patients hospitalized for ADHF would be defined specifically in this patient population. In the case of β-blocker treatment, the heart failure guidelines recommend its early induction in patients with ADHF and reduced LVEF, although the recommendation was based on small studies showing the association between withdrawal of β-blocker therapy at admission for ADHF and an increase in mortality.^16,17,18,19^

In the present study, MRA treatment was not associated with a lower risk for all-cause death but instead with heart failure hospitalization only in contemporary patients with ADHF who were discharged alive. To our knowledge, the baseline patient characteristics were substantially different from those in previous RCTs. A large proportion of patients with advanced age (median age of 80 years) and a substantial proportion of patients with comorbidities, such as chronic kidney disease and AF or AFL, were represented in this study. Despite these demographic differences, the favorable association of MRA with heart failure hospitalization but not with mortality in patients with HFpEF was consistent with findings reported in previous RCTs.

The mechanisms by which MRA may become potentially advantageous to patients with HFpEF from the point of heart failure readmission rate are uncertain. However, the neurohormonal pathways were excessively activated in a large proportion of patients with ADHF through sympathetic nerve systems, worsening renal function, and the aggressive use of loop diuretics.^20,21,22^ The use of angiotensin-converting enzyme inhibitor or angiotensin receptor blocker was less common in HFpEF than in HFrEF. Therefore, the blockade of the renin-angiotensin system by MRA might have promoted the favorable association with heart failure hospitalization in patients with HFpEF. Another possible mechanism was the diuretic nature of MRA. In this study, the combination of MRA and loop diuretics was more frequently observed in patients with HFpEF. Long-term use of loop diuretics is associated with diuretic resistance through tubule-glomerular feedback mechanisms enhanced by renin-angiotensin-aldosterone system activation.^20,21,22^ The blockade of aldosterone statistically significantly enhances the blockade of sodium reabsorption in the distal tubules and collecting duct. These speculated mechanisms were consistent with the observed favorable associations of MRA use with the heart failure readmission in patients with HFpEF.

However, no difference in all-cause mortality or any unexpected hospitalization was observed regardless of MRA use. One reason for this finding might be that many patients, particularly those with HFpEF, had many comorbidities owing to their advanced age. Thus, the outcomes of MRA may be counterbalanced by electrolyte imbalance, worsening renal function, and noncardiovascular mortality or hospitalization in patients who received MRA. Another consideration is that patients treated with MRA in the entire population were hypotensive, more likely to have reduced LVEF, and less likely to have ADHF from hypertensive heart disease. Therefore, the adverse effects of MRA use may have been avoided because the heart failure stage in the MRA use group was more progressive than the no MRA use group, although we performed the propensity score matching using 16 variables.

The lack of mortality advantage from MRA use despite a reduction in heart failure hospitalization was consistent with findings of an observational study in patients with ADHF and the TOPCAT trial.^3,5,15^ The therapy to reduce heart failure hospital readmissions has an important role in daily practice in a rapidly aging society because repeated heart failure hospitalizations are associated with high mortality rates, diminished quality of life and functional status, inadequate recovery after heart failure deterioration, and the escalation of the medical costs.^23^ In contrast, the rate of any unexpected hospitalization did not differ between the MRA use group and no MRA use group in the propensity score–matched cohort. Considering no differences in mortality or overall rate of hospitalization, MRA use may be associated with minimal, if any, clinical net advantages. Our additional analyses suggested the potential value of MRA use for patients with HFpEF. Exploratory studies to identify the patient groups that find MRA use advantageous are needed and should be confirmed by RCTs in patients hospitalized for ADHF.

### Limitations

This study has several limitations. First, the observational study design is subject to selection bias and residual confounding. The KCHF registry had comprehensive data on patient demographics, medical history, underlying heart disease, prehospital activities, socioeconomic status, signs, symptoms, medications, laboratory tests, electrocardiogram, and echocardiography results, acute management in the emergency department, status at discharge, and clinical events during the index hospitalization. By adjusting for 25 variables, we accounted for most conceivable confounders. Nevertheless, residual unmeasured confounding could affect the results. Second, we had no prescription data after discharge from the index hospitalization; therefore, we could not deny the possibility of substantial crossover in MRA use. Third, this observational study used propensity score matching. Thus, we did not perform a confidence set or power analysis in advance. Further studies, including those on quality of life and cost, may clarify the clinical net advantage of MRA use in this cohort. Fourth, data on the reasons for MRA use or no MRA use by individual patients were not available.

## Conclusions

Use of MRA at discharge from ADHF hospitalization appeared to be associated with a lower risk of heart failure hospitalization but not with lower all-cause mortality or overall rate of hospitalization. These findings suggest that MRA use might be associated with minimal, if any, clinical advantage. Further studies appear to be needed to identify the patient groups that may find value in MRA treatment and for findings to be confirmed by RCTs in patients hospitalized for ADHF.

## Notes

Supplement. **eTable 1.** Number of Missing Values in the Matched and Entire Cohort **eTable 2.** The Prescription of ACEI/ARB and Beta-blocker at Discharge in Patients With Reduced LVEF in the Matched and Entire Cohort **eTable 3.** The Prescription of ACEI/ARB and Beta-blocker at Discharge in Patients With Preserved LVEF in the Matched and Entire Cohort **eTable 4.** Association Between the Prescription of MRA at Discharge and Clinical Outcomes by the LVEF Categories in the Entire Cohort **eTable 5.** Patient Characteristics Before and After Propensity Score Matching in Reduced LVEF **eTable 6.** Patient Characteristics Before and After Propensity Score Matching in Preserved LVEF **eFigure 1.** Cumulative Incidence Function Curves of the MRA and No MRA Groups for HF Hospitalization **eFigure 2.** Cumulative Incidences of the Primary Outcome Measure (Death or HF Hospitalization) by LVEF in the Propensity Score-Matched Cohort **eFigure 3.** Cumulative Incidences of the Primary Outcome Measure (Death or HF Hospitalization) (A) All-Cause Death (B) and HF Hospitalization (C) in the Entire Cohort **eFigure 4.** Cumulative Incidences of the Primary Outcome Measure (Death or HF Hospitalization) (A) All-Cause Death (B) and HF Hospitalization (C) by LVEF in the Entire Cohort **eFigure 5.** Flowchart of the Propensity Score-Matched Cohort in Each LVEF Strata; <40% and ≥40% **eFigure 6.** Cumulative Incidences of the Primary Outcome Measure (Death or HF Hospitalization) in the Propensity Score-Matched Cohort in Each LVEF Strata
