# Supplementary material for: Association of Mineralocorticoid Receptor Antagonist Use With All-Cause Mortality and Hospital Readmission in Older Adults With Acute Decompensated Heart Failure
Source: JAMA Netw Open. 2019 Jun 21;2(6):e195892. doi: 10.1001/jamanetworkopen.2019.5892 (PMC6593642; doi:10.1001/jamanetworkopen.2019.5892)
Supplement: Supplement. — eTable 1. Number of Missing Values in the Matched and Entire Cohort eTable 2. The Prescription of ACEI/ARB and Beta-blocker at Discharge in Patients With Reduced LVEF in the Matched and Entire Cohort eTable 3. The Prescription of ACEI/ARB and Beta-blocker at Discharge in Patients With Preserved LVEF in the Matched and Entire Cohort eTable 4. Association Between the Prescription of MRA at Discharge and Clinical Outcomes by the LVEF Categories in the Entire Cohort eTable 5. Patient Characteristics Before and After Propensity Score Matching in Reduced LVEF eTable 6. Patient Characteristics Before and After Propensity Score Matching in Preserved LVEF eFigure 1. Cumulative Incidence Function Curves of the MRA and No MRA Groups for HF Hospitalization eFigure 2. Cumulative Incidences of the Primary Outcome Measure (Death or HF Hospitalization) by LVEF in the Propensity Score-Matched Cohort eFigure 3. Cumulative Incidences of the Primary Outcome Measure (Death or HF Hospitalization) (A) All-Cause Death (B) and HF Hospitalization (C) in the Entire Cohort eFigure 4. Cumulative Incidences of the Primary Outcome Measure (Death or HF Hospitalization) (A) All-Cause Death (B) and HF Hospitalization (C) by LVEF in the Entire Cohort eFigure 5. Flowchart of the Propensity Score-Matched Cohort in Each LVEF Strata; <40% and ≥40% eFigure 6. Cumulative Incidences of the Primary Outcome Measure (Death or HF Hospitalization) in the Propensity Score-Matched Cohort in Each LVEF Strata [file jamanetwopen-2-e195892-s001.pdf]

Supplementary Online Content

Yaku H, Kato T, Morimoto T, et al; KCHF Study Investigators. Association of mineralocorticoid receptor antagonist use with all-cause mortality and hospital readmission in older adults with acute decompensated heart failure. *JAMA Netw Open.* 2019;2(6):e195892. doi:10.1001/jamanetworkopen.2019.5892

eTable 1. Number of Missing Values in the Matched and Entire Cohort
eTable 2. The Prescription of ACEI/ARB and Beta-blocker at Discharge in Patients With Reduced LVEF in the Matched and Entire Cohort
eTable 3. The Prescription of ACEI/ARB and Beta-blocker at Discharge in Patients With Preserved LVEF in the Matched and Entire Cohort
eTable 4. Association Between the Prescription of MRA at Discharge and Clinical Outcomes by the LVEF Categories in the Entire Cohort
eTable 5. Patient Characteristics Before and After Propensity Score Matching in Reduced LVEF
eTable 6. Patient Characteristics Before and After Propensity Score Matching in Preserved LVEF
eFigure 1. Cumulative Incidence Function Curves of the MRA and No MRA Groups for HF Hospitalization
eFigure 2. Cumulative Incidences of the Primary Outcome Measure (Death or HF Hospitalization) by LVEF in the Propensity Score-Matched Cohort
eFigure 3. Cumulative Incidences of the Primary Outcome Measure (Death or HF Hospitalization) (A) All-Cause Death (B) and HF Hospitalization (C) in the Entire Cohort
eFigure 4. Cumulative Incidences of the Primary Outcome Measure (Death or HF Hospitalization) (A) All-Cause Death (B) and HF Hospitalization (C) by LVEF in the Entire Cohort
eFigure 5. Flowchart of the Propensity Score-Matched Cohort in Each LVEF Strata; <40% and ≥40%
eFigure 6. Cumulative Incidences of the Primary Outcome Measure (Death or HF Hospitalization) in the Propensity Score-Matched Cohort in Each LVEF Strata

This supplementary material has been provided by the authors to give readers additional information about their work.

©2019 Yaku H et al. *JAMA Network Open*

eTable 1. Number of Missing Values in the Matched and Entire Cohort

	Matched Cohort (N=2068)			Entire Cohort (N=3717)	
	Number	Rate (%)		Number	Rate (%)
Clinical characteristics					
Body mass index	89	4.30		174	4.68
Daily life activities	14	0.68		37	1.00
Tests at admission					
BNP or NT-proBNP *	21	1.02		46	1.24
Serum creatinine	0	0		6	0.16
eGFR	0	0		6	0.16
Blood urea nitrogen	1	0.05		11	0.30
Albumin	55	2.66		110	2.96
Sodium	0	0		13	0.35
Potassium	0	0		13	0.35
Hemoglobin	0	0		6	0.16

* BNP values were reported for 1887 patients in the matched cohort and 3394 patients in the entire cohort; NT-proBNP values were reported for 181 patients in the matched cohort and 323 patients in the entire cohort.

Abbreviation: BNP = brain-type natriuretic peptide; NT-proBNP = N-terminal-proBNP; eGFR = estimated glomerular filtration rate.

©2019 Yaku H et al. *JAMA Network Open*

eTable 2. The Prescription of ACEI/ARB and Beta-blocker at Discharge in Patients With Reduced LVEF in the Matched and Entire Cohort

	Matched Cohort				Entire Cohort		
	MRA Use (N=368)	No MRA Use (N=370)	P Value		MRA Use (N=722)	No MRA Use (N=661)	P Value
Medications at discharge							
ACEI/ARB and Beta-blocker	221 (60)	203 (55)	0.15		456 (63)	299 (45)	<0.001
ACEI/ARB	255 (69)	236 (64)	0.11		517 (72)	375 (57)	<0.001
Beta-blocker	295 (80)	291(79)	0.61		607 (84)	473 (72)	<0.001

ACEI = angiotensin-converting enzyme inhibitor; ARB = angiotensin-receptor blocker; LVEF = left ventricular ejection fraction; MRA = mineralocorticoid receptor antagonist.

©2019 Yaku H et al. *JAMA Network Open*

eTable 3. The Prescription of ACEI/ARB and Beta-blocker at Discharge in Patients With Preserved LVEF in the Matched and Entire Cohort

	Matched Cohort				Entire Cohort		
	MRA Use (N=666)	No MRA Use (N=664)	P Value		MRA Use (N=956)	No MRA Use (N=1378)	P Value
Medications at discharge							
ACEI/ARB and Beta-blocker	243 (36)	260 (39)	0.32		363 (38)	443 (32)	0.004
ACEI/ARB	370 (56)	391 (59)	0.22		534 (56)	712 (52)	0.046
Beta-blocker	407 (61)	412 (62)	0.73		596 (62)	793 (58)	0.02

ACEI = angiotensin-converting enzyme inhibitor; ARB = angiotensin-receptor blocker; LVEF = left ventricular ejection fraction; MRA = mineralocorticoid receptor antagonist.

©2019 Yaku H et al. *JAMA Network Open*

eTable 4. Association Between the Prescription of MRA at Discharge and Clinical Outcomes by the LVEF Categories in the Entire Cohort

		Unadjusted		Adjusted		
		**HR* (95%CI)**	**P Value**	**HR* (95%CI)**	**P Value**	**P Value for interaction**
**Primary outcome measure**						
A composite of all-cause death or HF hospitalization	HFrEF	0.76 (0.64–0.89)	0.001	0.92 (0.75–1.15)	0.47	0.10
	HFpEF	0.80 (0.70–0.91)	<0.001	0.73 (0.62–0.87)	<0.001	
**Secondary outcome measures**						
HF hospitalization	HFrEF	0.73 (0.60–0.89)	0.002	0.83 (0.64–1.08)	0.17	0.28
	HFpEF	0.77 (0.65–0.92)	0.003	0.68 (0.55–0.84)	<0.001	
All-cause death	HFrEF	0.80 (0.64–1.01)	0.06	1.10 (0.82–1.47)	0.53	0.33
	HFpEF	0.85 (0.71–1.01)	0.06	0.85 (0.68–1.05)	0.14	

Values are N (%) unless otherwise indicated. *The HR and 95% CI of the unadjusted and adjusted models for one-year outcomes according to MRA treatment at discharge.

MRA = mineralocorticoid receptor antagonist; LVEF = left ventricular ejection fraction; HR = hazard ratio; CI = confidence interval; HF = heart failure; HFrEF = heart failure with reduced ejection fraction (<40%); HFpEF = heart failure with preserved ejection fraction (≥40%).

©2019 Yaku H et al. *JAMA Network Open*

eTable 5. Patient Characteristics Before and After Propensity Score Matching in Reduced LVEF

	Before propensity score matching					After propensity score matching				
	MRA Use (N=722)		No MRA Use (N=661)		P Value	MRA Use (N=385)		No MRA Use (N=385)		P Value
Clinical characteristics										
Age, y	75	(63–83)	78	(69–85)	<0.001	77	(66–84)	77	(68–85)	0.61
Age ≥80 y*	252	(35)	297	(45)	<0.001	159	(41)	151	(39)	0.56
Female sex*	244	(34)	214	(32)	0.58	127	(33)	127	(33)	1.00
BMI, kg/m^2	23.2 ± 5.0		22.5	± 4.2	0.007	22.8	± 4.6	22.8	± 4.4	0.90
BMI ≤22 kg/m^2	314	(45)	314	(49)	0.17	186	(50)	174	(47)	0.31
Origin										
Ischemic heart disease	295	(41)	325	(49)	0.002	178	(46)	178	(46)	1.00
ACS	35	(4.8)	51	(7.7)	0.03	26	(6.8)	30	(7.8)	0.58
Hypertensive heart disease	87	(12)	97	(15)	0.15	45	(12)	55	(14)	0.28
Cardiomyopathy	252	(35)	145	(22)	<0.001	120	(31)	108	(28)	0.34
Valvular heart disease	64	(8.9)	70	(11)	0.28	31	(8.1)	34	(8.8)	0.70
Medical history										
Previous HF hospitalization*	257	(36)	274	(43)	0.02	131	(35)	129	(35)	1.00
Atrial fibrillation or flutter	229	(32)	209	(32)	0.97	114	(30)	126	(33)	0.35
Hypertension*	444	(62)	467	(71)	<0.001	244	(63)	249	(65)	0.71

©2019 Yaku H et al. *JAMA Network Open*

Diabetes*	277	(38)	290	(44)	0.04		150	(39)	162	(42)	0.38
Dyslipidemia	287	(40)	295	(45)	0.07		163	(43)	164	(43)	0.94
Previous myocardial infarction*	204	(28)	238	(36)	0.002		124	(32)	122	(32)	0.88
Previous stroke	82	(11)	108	(16)	0.007		45	(12)	52	(14)	0.45
Previous PCI or CABG	203	(28)	231	(35)	0.006		124	(32)	114	(30)	0.44
Current smoking	134	(19)	87	(13)	0.007		60	(16)	57	(15)	0.79
VT or VF	63	(8.7)	52	(7.9)	0.56		30	(7.8)	35	(9.1)	0.52
Chronic lung disease	81	(11)	89	(14)	0.20		40	(10)	53	(14)	0.15
Malignancy	89	(12)	91	(14)	0.43		51	(13)	51	(13)	1.00
Dementia	99	(14)	106	(16)	0.22		63	(16)	61	(16)	0.85
Social backgrounds											
With Occupation	162	(22)	109	(17)	0.005		75	(20)	72	(19)	0.78
Living alone	178	(25)	130	(20)	0.03		98	(26)	80	(21)	0.12
Daily life activities											
Ambulatory	607	(85)	537	(82)	0.12		318	(84)	316	(83)	0.79
Use of wheelchair	83	(12)	93	(14)	0.16		49	(13)	52	(14)	0.76
Bedridden	23	(3.2)	25	(3.8)	0.55		14	(3.7)	14	(3.7)	0.99
Vital signs at presentation											
BP, mmHg											
Systolic BP	141	± 33	145	± 34	0.02		143	± 33	143	± 33	0.85
Systolic BP <90*	25	(3.5)	16	(2.4)	0.25		11	(2.9)	7	(1.8)	0.34

©2019 Yaku H et al. *JAMA Network Open*

Diastolic BP, mean (SD)	89	(24)	88	(24)	0.64		89	(23)	89	(23)	0.95
Heart rate, bpm	101	(25)	101	(26)	0.68		101	(23)	103	(27)	0.16
Heart rate <60 bpm	17	(2.4)	17	(2.6)	0.78		7	(1.8)	7	(1.8)	1.00
NYHA Class III or IV*	629	(88)	584	(89)	0.46		328	(86)	341	(89)	0.16
Tests at admission											
LVEF, %	29	± 7.1	30	± 7.1	0.01		29	± 7.3	29	± 7.3	0.49
BNP, pg/mL	922	(579–21556)	1013	(581–1736)	0.08		946	(599–1600)	929	(562–1615)	0.81
NT-proBNP, pg/mL	5186	(2843–13579)	7581	(4613–16796)	0.02		7048	(2568–11632)	6325	(2917–9345)	0.79
Serum creatinine, mg/dL	1.0	(0.8–1.3)	1.4	(1.0–2.1)	<0.001		1.0	(0.8–1.4)	1.1	(0.87–1.46)	0.03
eGFR, mL/min/1.73m^2	53	(39–67)	37	(24–54)	<0.001		51	(35–65)	45	(35–60)	0.01
eGFR <30 mL/min/1.73m^2*	93	(13)	244	(37)	<0.001		68	(18)	52	(14)	0.12
Blood urea nitrogen, mg/dL	21	(16–29)	27	(20– 42)	<0.001		22	(17–30)	23	(17–32)	0.37
Albumin, g/dL	3.6	± 0.5	3.5	± 0.5	0.02		3.5	± 0.5	3.5	± 0.5	0.69
Albumin <3.0 g/dL	68	(9.7)	81	(13)	0.09		36	± 9.6	35	± 9.4	0.90
Sodium, mEq/L	139	± 4.2	139	± 4.1	0.21		139	± 4.3	139	± 4.0	0.11
Sodium <135 mEq/L	102	(14)	64	(9.8)	0.01		57	(15)	32	(8.4)	0.005
Potassium, mEq/L	4.1	± 0.6	4.3	± 0.7	<0.001		4.1	± 0.7	4.2	± 0.6	0.04
Potassium ≥5.0 mEq/L*	69	(9.6)	101	(15)	0.001		37	(9.6)	43	(11)	0.46
Hemoglobin, g/dL	12.8	± 2.3	11.7	± 2.4	<0.001		12.5	± 2.4	12.3	± 2.4	0.21
Anemia*	336	(47)	430	(65)	<0.001		207	(54)	204	(53)	0.89
MRA before the index admission*	237	(33)	48	(7.3)	<0.001		51	(13)	45	(12)	0.51

©2019 Yaku H et al. *JAMA Network Open*

Medications at discharge											
ACEI or ARB*	517	(72)	375	(57)	<0.001		254	(66)	249	(65)	0.71
Beta-blocker*	607	(84)	473	(72)	<0.001		303	(79)	299	(78)	0.73
Loop diuretics*	660	(91)	486	(74)	<0.001		327	(85)	324	(84)	0.77
Thiazide	29	(4.0)	34	(5.1)	0.32		11	(2.9)	16	(4.2)	0.33
Tolvaptan	82	(11)	87	(13)	0.31		46	(12)	41	(11)	0.57
Digoxin	54	(7.5)	34	(5.1)	0.08		18	(4.7)	27	(7.0)	0.17
Warfarin sodium	170	(24)	159	(24)	0.82		93	(24)	91	(24)	0.87
DOAC	129	(18)	98	(15)	0.13		57	(15)	72	(19)	0.15

Values are median (IQR), mean ± SD, or number (%).

* Variables relevant to the choice of MRA selected for logistic regression model to develop propensity score for the choice of MRA.

Abbreviation: LVEF = left ventricular ejection fraction; BMI = body mass index; PCI = percutaneous coronary intervention; CABG = coronary artery bypass graft; VT/VF = ventricular tachycardia/fibrillation; bpm = beat per minute; NYHA = New York Heart Association; BP = blood pressure; BNP = brain-type natriuretic peptide; NT-proBNP = N-terminal-proBNP; eGFR = estimated glomerular filtration rate; ACEI = angiotensin-converting enzyme inhibitor; ARB = angiotensin-receptor blocker; DOAC = direct oral anticoagulant.

©2019 Yaku H et al. *JAMA Network Open*

eTable 6. Patient Characteristics Before and After Propensity Score Matching in Preserved LVEF

	Before propensity score matching						After propensity score matching				
	MRA						MRA				
	Received (N=956)		Not Received (N=1378)		P Value		Received (N=661)		Not Received (N=661)		P Value
Clinical characteristics											
Age, y	81	(74–87)	82	(75–87)	0.09		82	(74–87)	82	(75–87)	0.73
Age ≥80 y*	541	(57)	838	(61)	0.04		398	(58)	400	(58)	0.91
Female sex*	519	(54)	691	(50)	0.049		369	(54)	355	(51)	0.45
BMI, kg/m^2	23	± 4.6	23	± 4.2	0.58		22.9	± 4.5	22.9	± 4.0	0.90
BMI ≤22 kg/m^2	430	(48)	580	(44)	0.09		307	(47)	291	(44)	0.26
Origin											
Ischemic heart disease	235	(25)	350	(25)	0.65		176	(26)	152	(22)	0.13
ACS	51	(5.3)	68	(4.9)	0.67		44	(6.4)	25	(3.6)	0.02
Hypertensive heart disease	281	(29)	462	(34)	0.04		221	(32)	220	(32)	0.95
Cardiomyopathy	66	(6.9)	93	(6.7)	0.88		42	(6.1)	56	(8.1)	0.14
Valvular heart disease	274	(29)	327	(24)	0.007		176	(26)	182	(26)	0.71
Medical history											
Previous HF hospitalization*	292	(31)	494	(37)	0.005		186	(27)	196	(29)	0.48
Atrial fibrillation or flutter	485	(51)	627	(46)	0.01		319	(46)	331	(48)	0.52

©2019 Yaku H et al. *JAMA Network Open*

Hypertension*	691	(72)	1088	(79)	<0.001		529	(77)	533	(77)	0.80
Diabetes*	318	(33)	507	(37)	0.08		230	(33)	221	(32)	0.61
Dyslipidemia	325	(34)	545	(40)	0.006		231	(34)	267	(39)	0.04
Previous myocardial infarction*	162	(17)	232	(17)	0.95		112	(16)	121	(18)	0.52
Previous stroke	166	(17)	234	(17)	0.81		123	(18)	105	(15)	0.19
Previous PCI or CABG	189	(20)	330	(24)	0.02		139	(20)	150	(22)	0.47
Current smoking	88	(9.4)	143	(11)	0.34		66	(9.8)	76	(11)	0.40
VT or VF	23	(2.4)	16	(1.2)	0.02		13	(1.9)	8	(1.2)	0.27
Chronic lung disease	122	(13)	196	(14)	0.31		87	(13)	91	(13)	0.75
Malignancy	145	(15)	210	(15)	0.96		115	(17)	103	(15)	0.38
Dementia	182	(19)	268	(19)	0.81		132	(19)	132	(19)	1.00
Social backgrounds											
With Occupation	99	(10)	124	(9.0)	0.27		73	(11)	66	(9.6)	0.53
Living alone	196	(21)	290	(21)	0.75		138	(20)	140	(20)	0.89
Daily life activities											
Ambulatory	745	(79)	1052	(77)	0.41		553	(81)	545	(80)	0.66
Use of wheelchair	167	(18)	267	(20)	0.24		106	(16)	118	(17)	0.37
Bedridden	36	(3.8)	45	(3.3)	0.52		26	(3.8)	20	(2.9)	0.37
Vital signs at presentation											
BP, mmHg											
Systolic BP	148	± 34	154	± 36	<0.001		151	± 35	153	± 36	0.29

©2019 Yaku H et al. *JAMA Network Open*

Systolic BP <90*	23	(2.4)	31	(2.3)	0.81		15	(2.2)	22	(3.2)	0.24
Diastolic BP, mmHg	83	(23)	84	(24)	0.67		84	(23)	84	(24)	0.93
Heart rate, bpm	94	± 28	92	± 29	0.046		96	± 28	94	± 29	0.28
Heart rate <60 bpm	70	(7.4)	146	(11)	0.007		47	(6.9)	61	(8.9)	0.16
NYHA Class III or IV*	827	(87)	1182	(86)	0.37		594	(87)	600	(87)	0.84
Tests at admission											
LVEF, %	56	± 10	57	± 10	0.006		56	± 10	57	± 11	0.06
BNP, pg/mL	520	(295–942)	605	(336–1066)	0.003		525	(310–952)	580	(329–973)	0.56
NT-proBNP, pg/mL	4546	(2281–8915)	5597	(2698–13246)	0.02		4640	(2282–9345)	5151	(3214–9988)	0.30
Serum creatinine, mg/dL	1.0	(0.74–1.29)	1.2	(0.9–1.8)	<0.001		1.0	(0.7–1.3)	1.1	(0.8–1.4)	<0.001
eGFR, mL/min/1.73m^2	50	(36–66)	38	(24–55)	<0.001		50	(35–67)	45	(33–59)	<0.001
eGFR <30 mL/min/1.73m^2*	160	(17)	481	(35)	<0.001		128	(19)	127	(18)	0.93
Blood urea nitrogen, mg/dL	21	(16–29)	26	(19–38)	<0.001		21	(16–29)	23	(17–31)	<0.001
Albumin, g/dL	3.5	± 0.5	3.5	± 0.5	0.79		3.5	± 0.5	3.5	± 0.5	0.83
Albumin <3.0 g/dL	142	(15)	189	(14)	0.41		100	(15)	89	(13)	0.38
Sodium, mEq/L	139	± 4.4	139	± 4.1	0.58		139	± 4.3	140	± 3.8	0.052
Sodium <135 mEq/L	120	(13)	147	(11)	0.16		79	(12)	61	(8.9)	0.107
Potassium, mEq/L	4.1	± 0.6	4.3	± 0.7	<0.001		4.1	± 0.7	4.2	± 0.6	0.01
Potassium ≥5.0 mEq/L*	66	(6.9)	196	(14)	<0.001		56	(8.1)	63	(9.1)	0.51
Hemoglobin, g/dL	11	± 2.2	11	± 2.2	0.001		11.4	± 2.2	11.3	± 2.2	0.40
Anemia*	659	(69)	1032	(75)	0.001		475	(69)	471	(68)	0.82

©2019 Yaku H et al. *JAMA Network Open*

MRA before the index admission*	285	(30)	81	(5.9)	<0.001		77	(11)	78	(11)	0.93
Medications at discharge											
ACEI or ARB*	534	(56)	712	(52)	0.046		391	(57)	396	(57)	0.79
Beta-blocker*	596	(62)	793	(58)	0.02		424	(61)	424	(61)	1.00
Loop diuretics*	881	(92)	988	(72)	<0.001		617	(89)	614	(89)	0.80
Thiazide	44	(4.6)	111	(8.1)	0.001		31	(4.5)	59	(8.6)	0.002
Tolvaptan	94	(9.8)	127	(9.2)	0.62		57	(8.3)	59	(8.6)	0.85
Digoxin	73	(7.6)	50	(3.6)	<0.001		43	(6.2)	37	(5.4)	0.49
Warfarin sodium	250	(26)	345	(25)	0.54		156	(23)	165	(24)	0.57
DOAC	250	(26)	283	(21)	0.001		190	(28)	179	(26)	0.50

Values are median (IQR), mean ± SD, or number (%).

* Variables relevant to the choice of MRA selected for logistic regression model to develop propensity score for the choice of MRA.

Abbreviation: LVEF = left ventricular ejection fraction; BMI = body mass index; PCI = percutaneous coronary intervention; CABG = coronary artery bypass graft; VT/VF = ventricular tachycardia/fibrillation; bpm = beat per minute; NYHA = New York Heart Association; BP = blood pressure; BNP = brain-type natriuretic peptide; NT-proBNP = N-terminal-proBNP; eGFR = estimated glomerular filtration rate; ACEI = angiotensin-converting enzyme inhibitor; ARB = angiotensin-receptor blocker; DOAC = direct oral anticoagulant.

©2019 Yaku H et al. *JAMA Network Open*

eFigure 1. Cumulative Incidence Function Curves of the MRA and No MRA Groups for HF Hospitalization

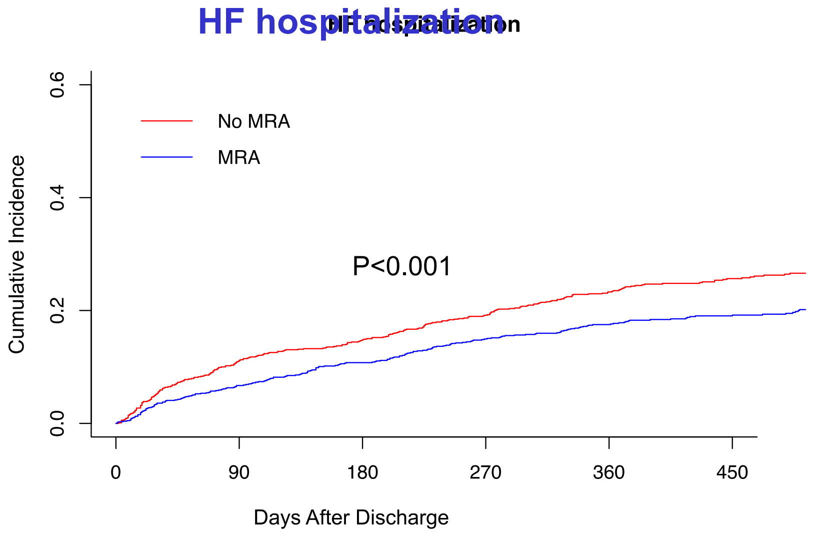

Interval	90 Days	180 Days	1 Year
**No MRA**			
Cumulative incidence [upper–lower 95% CI]	5.3% [4.1–6.8%]	14.8% [12.7–17.1%]	23.5% [21.0–26.2%]
**MRA**			
Cumulative incidence [upper–lower 95% CI]	3.6% [2.6–4.9%]	10.8% [9.0–12.8%]	17.7% [15.4–20.1%]

MRA = mineralocorticoid receptor antagonist; HF = heart failure.

eFigure 2. Cumulative Incidences of the Primary Outcome Measure (Death or HF Hospitalization) by LVEF in the Propensity Score-Matched Cohort

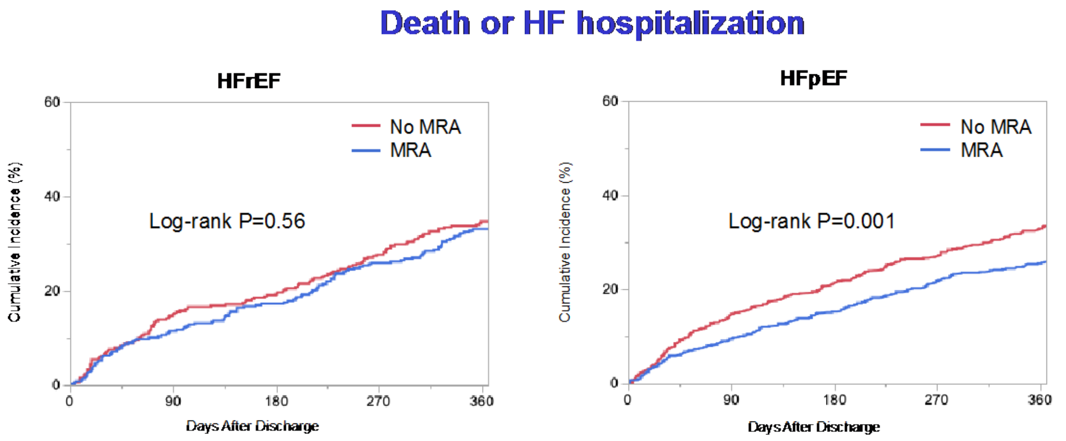

HFrEF

Interval	0 Day	30 Days	180 Days	1 Year
No MRA				
N of patients with at least one event		24	71	126
N of patients at risk	370	346	293	203
Cumulative incidence		6.5%	19.1%	34.7%
MRA				
N of patients with at least one event		23	63	119
N of patients at risk	368	345	297	212
Cumulative incidence		6.3%	17.2%	33.1%

HFpEF

Interval	0 Day	30 Days	180 Days	1 Year
No MRA				
N of patients with at least one event		39	141	218
N of patients at risk	664	621	514	386
Cumulative incidence		5.9%	21.4%	33.4%
MRA				
N of patients with at least one event		31	100	168
N of patients at risk	666	634	549	444
Cumulative incidence		4.7%	16.0%	25.9%

HF = heart failure; LVEF = left ventricular ejection fraction; HFrEF = heart failure with reduced ejection fraction (<40%); HFpEF = heart failure with preserved ejection fraction (≥40%); MRA = mineralocorticoid receptor antagonist.

eFigure 3. Cumulative Incidences of the Primary Outcome Measure (Death or HF Hospitalization) (A) All-cause Death (B) and HF Hospitalization (C) in the Entire Cohort

(A)

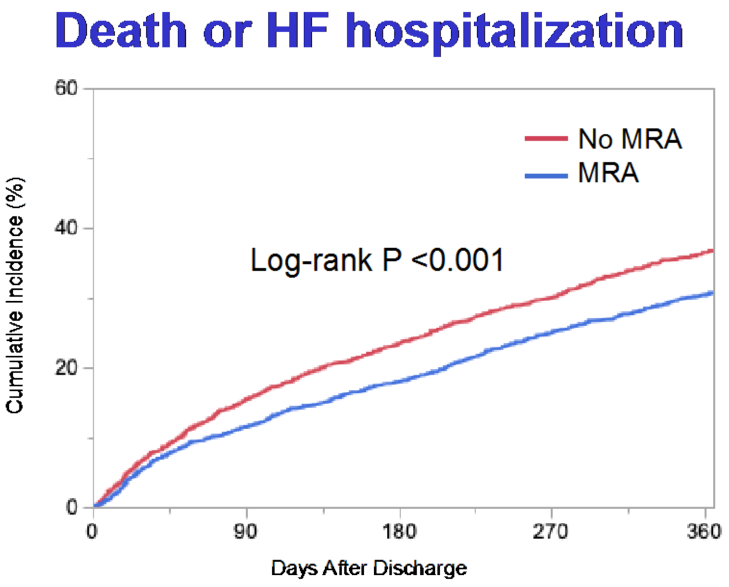

Interval	0 Day	30 Days	180 Days	1 Year
**No MRA**				
Patients with at Least One Event		140	476	739
Patients at Risk	2039	1894	1539	1134
Cumulative incidence		6.9%	23.5%	36.8%
**MRA**				
Patients with at Least One Event		95	300	503
Patients at Risk	1678	1579	1343	1020
Cumulative incidence		5.7%	18.1%	30.6%

©2019 Yaku H et al. *JAMA Network Open*

(B)

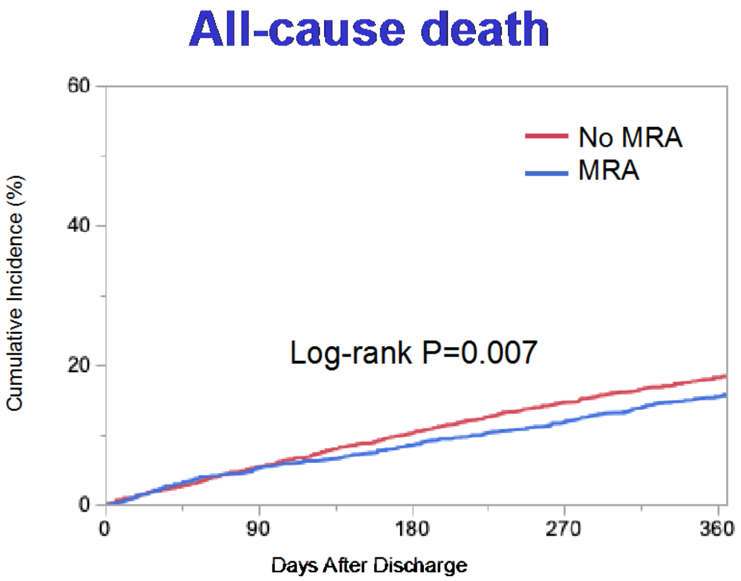

Intervalwe	0 Day	30 Days	180 Days	1 Year
**No MRA**				
N of patients with event		40	207	367
N of patients at risk	2039	1989	1794	1461
Cumulative incidence		2.0%	10.3%	18.4%
**MRA**				
N of patients with event		34	142	257
N of patients at risk	1678	1637	1498	1229
Cumulative incidence		2.0%	8.6%	15.7%

©2019 Yaku H et al. *JAMA Network Open*

(C)

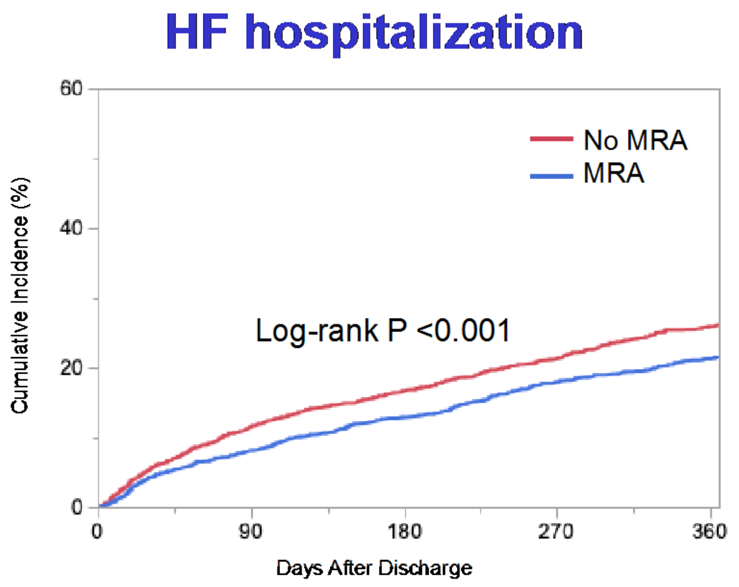

Interval	0 Day	30 Days	180 Days	1 Year
**No MRA**				
N of patients with at least one event		108	328	494
N of patients at risk	2039	1894	1539	1134
Cumulative incidence		5.4%	16.7%	26.2%
**MRA**				
N of patients with at least one event		71	208	335
N of patients at risk	1678	1579	1343	1020
Cumulative incidence		4.3%	12.9%	21.5%

HF = heart failure; MRA = mineralocorticoid receptor antagonist.

©2019 Yaku H et al. *JAMA Network Open*

eFigure 4. Cumulative Incidences of the Primary Outcome Measure (Death or HF Hospitalization) (A) All-cause Death (B) and HF Hospitalization (C) by LVEF in the Entire Cohort

(A)

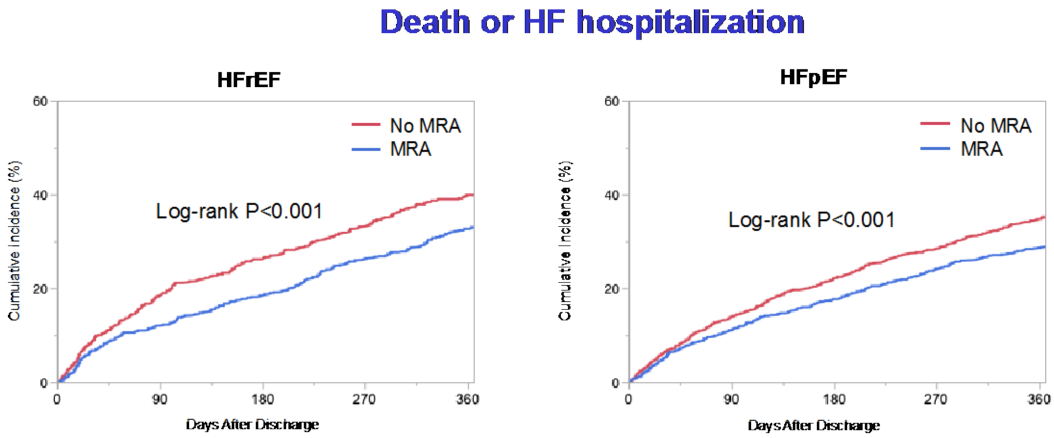

HFrEF

Interval	0 Day	30 Days	180 Days	1 Year
No MRA				
N of patients with at least one event		56	173	260
N of patients at risk	661	605	479	340
Cumulative incidence		8.5%	26.4%	39.9%
MRA				
N of patients with at least one event		48	133	233
N of patients at risk	722	673	576	412
Cumulative incidence		6.7%	18.6%	33.0%

HFpEF

Interval	0 Day	30 Days	180 Days	1 Year
No MRA				
N of patients with at least one event		84	303	479
N of patients at risk	1378	1289	1060	794
Cumulative incidence		6.1%	22.1%	35.3%
MRA				
N of patients with at least one event		47	167	270
N of patients at risk	956	906	767	608
Cumulative incidence		4.8%	17.7%	28.9%

©2019 Yaku H et al. *JAMA Network Open*

(B)

All-cause death

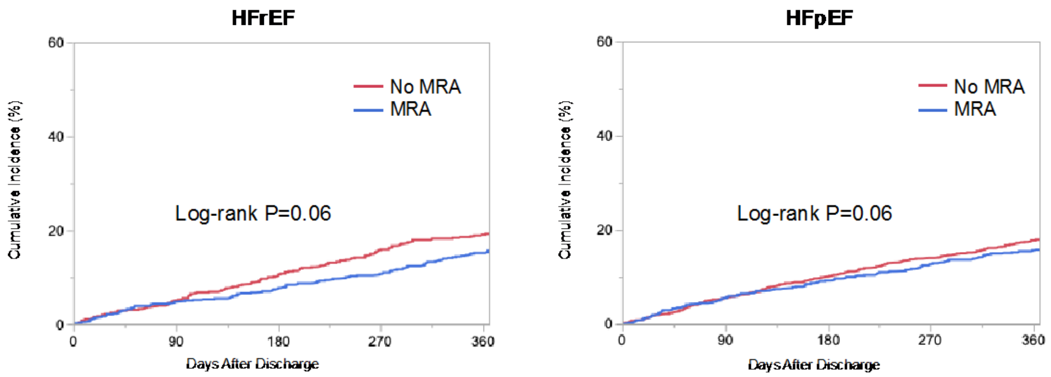

HFrEF

Interval	0 Day	30 Days	180 Days	1 Year
**No MRA**				
N of patients with event		14	69	125
N of patients at risk	661	643	576	455
Cumulative incidence		2.1%	10.6%	19.4%
**MRA**				
N of patients with event		15	55	110
N of patients at risk	722	705	653	515
Cumulative incidence		2.1%	7.8%	15.7%

HFpEF

Interval	0 Day	30 Days	180 Days	1 Year
**No MRA**				
N of patients with event		26	138	242
N of patients at risk	1378	1346	1218	1006
Cumulative incidence		1.9%	10.1%	17.9%
**MRA**				
N of patients with event		19	87	147
N of patients at risk	956	932	845	714
Cumulative incidence		2.0%	9.2%	15.8%

©2019 Yaku H et al. *JAMA Network Open*

(C)

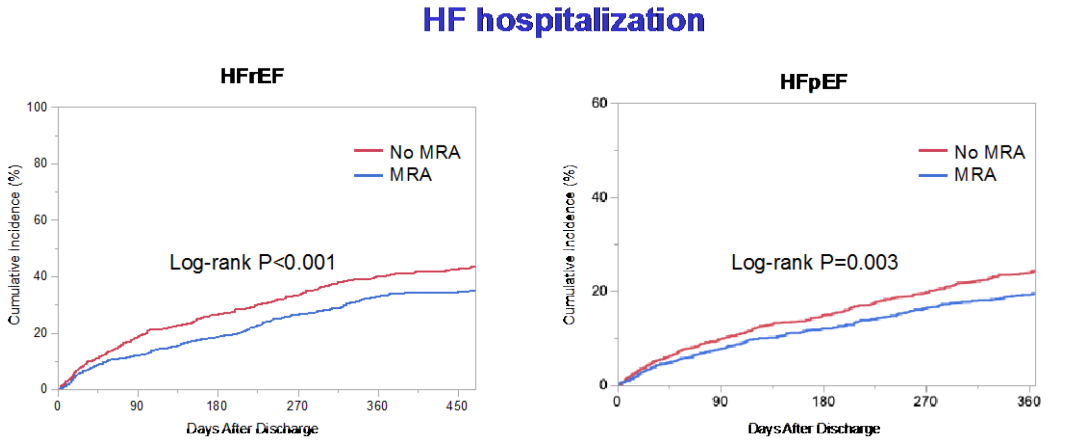

HFrEF

Interval	0 Day	30 Days	180 Days	1 Year
**No MRA**				
N of patients with at least one event		45	132	186
N of patients at risk	661	605	479	340
Cumulative incidence		6.9%	20.7%	30.2%
**MRA**				
N of patients with at least one event		36	98	163
N of patients at risk	722	673	576	412
Cumulative incidence		5.1%	14.1%	24.3%

HFpEF

Interval	0 Day	30 Days	180 Days	1 Year
**No MRA**				
N of patients with at least one event		63	196	308
N of patients at risk	1378	1289	1060	794
Cumulative incidence		4.6%	14.8%	24.3%
**MRA**				
N of patients with at least one event		35	110	172
N of patients at risk	956	906	767	608
Cumulative incidence		3.7%	12.0%	19.4%

HF = heart failure; LVEF = left ventricular ejection fraction; HFrEF = heart failure with reduced ejection fraction (<40%); HFpEF = heart failure with preserved ejection fraction (≥40%); MRA = mineralocorticoid receptor antagonist.

©2019 Yaku H et al. *JAMA Network Open*

eFigure 5. Flowchart of the Propensity Score-Matched Cohort in Each LVEF Strata; <40% and ≥40%

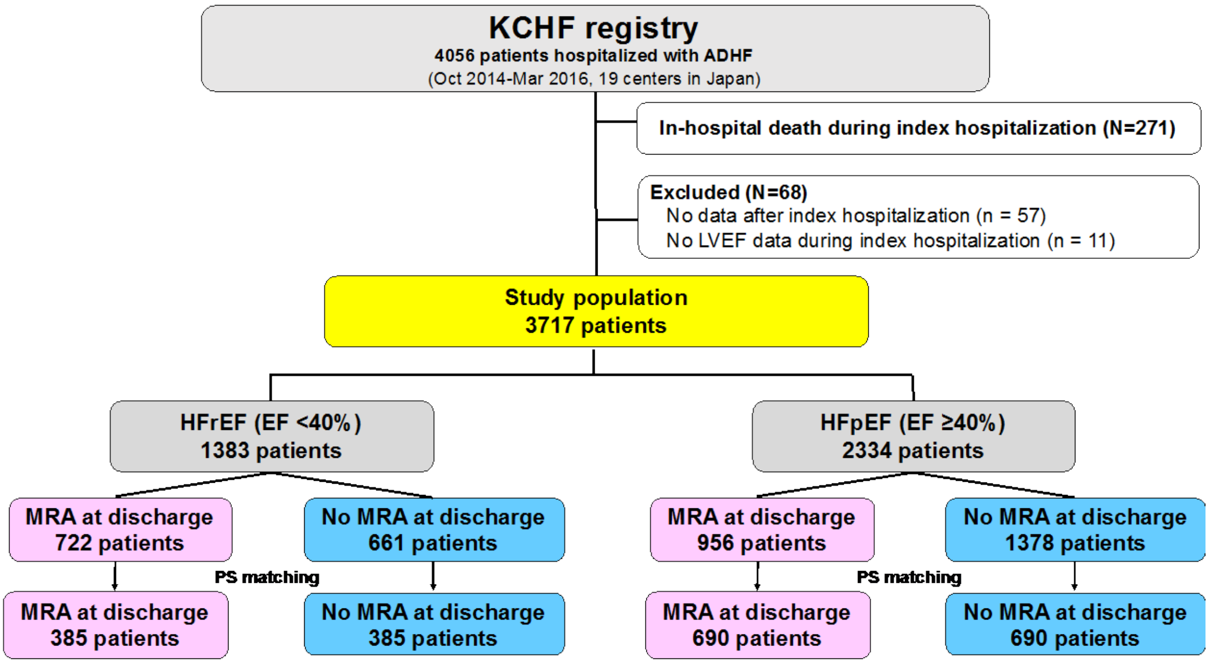

LVEF = left ventricular ejection fraction; HFrEF = heart failure with reduced ejection fraction; HFpEF = heart failure with preserved ejection fraction; MRA = mineralocorticoid receptor antagonist.

©2019 Yaku H et al. *JAMA Network Open*

eFigure 6. Cumulative Incidences of the Primary Outcome Measure (Death or HF Hospitalization) in the Propensity Score-Matched Cohort in Each LVEF Strata

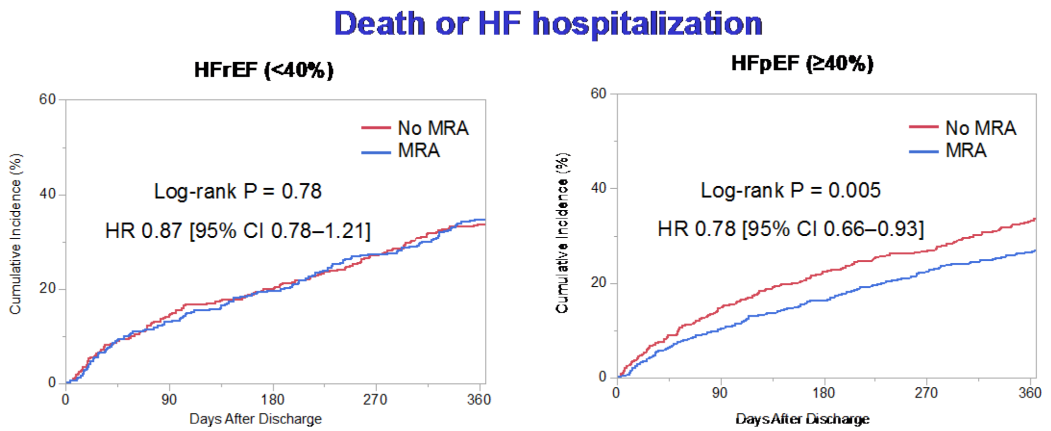

HFrEF (<40%)

Interval	0 Day	30 Days	180 Days	1 Year
**No MRA**				
Patients with at Least One Event		55	76	128
Patients at Risk	385	327	304	222
Cumulative incidence		14.4%	19.9%	33.7%
**MRA**				
Patients with at Least One Event		50	75	131
Patients at Risk	385	331	304	219
Cumulative incidence		13.0%	19.6%	34.7%

HFpEF (≥40%)

Interval	0 Day	30 Days	180 Days	1 Year
**No MRA**				
Patients with at Least One Event		102	153	229
Patients at Risk	690	584	529	399
Cumulative incidence		14.8%	22.3%	33.7%
**MRA**				
Patients with at Least One Event		69	111	181
Patients at Risk	690	608	563	450
Cumulative incidence		10.1%	16.3%	26.8%

HF = heart failure; LVEF = left ventricular ejection fraction; HFrEF = heart failure with reduced ejection fraction; HFpEF = heart failure with preserved ejection fraction; MRA = mineralocorticoid receptor antagonist.

©2019 Yaku H et al. *JAMA Network Open*
